# Prevalence of pale, soft, and exudative (PSE) condition in chicken meat used for commercial meat processing and its effect on roasted chicken breast

**DOI:** 10.1186/s40781-016-0110-8

**Published:** 2016-07-25

**Authors:** Deshani S. Karunanayaka, Dinesh D. Jayasena, Cheorun Jo

**Affiliations:** Department of Animal Science, Uva Wellassa University, Badulla, 90000 Sri Lanka; Department of Agricultural Biotechnology, Center for Food and Bioconvergence, and Research Institute of Agriculture and Life Sciences, Seoul National University, Seoul, 151-921 Republic of Korea

**Keywords:** Broiler meat, Color, Cooking loss, pH, PSE, Water holding capacity

## Abstract

**Background:**

Studies on prevalence of pale, soft, exudative (PSE) condition in Sri Lankan poultry industry is minimal. Hence, the objective of present study was to determine the incidence of PSE chicken meat in a commercial meat processing plant and to find out its consequences on meat quality traits of roasted chicken breast.

**Method:**

A total of 60 breast fillets were randomly selected, evaluated based on color L* value, and placed into 1 of 2 categories; PSE (L* > 58) or normal meat (L* ≤ 58). A total of 20 breast fillets (10 PSE and 10 normal) were then analyzed for color, pH, and water holding capacity (WHC). After processing those into roasted chicken breast, cooking loss, color, pH, WHC, and texture values were evaluated. A sensory evaluation was conducted using 30 untrained panelists.

**Results:**

The incidence of PSE meat was 70 % in the present experiment. PSE fillets were significantly lighter and had lower pH values compared with normal fillets. Correlation between the lightness and pH was negative (*P* < 0.05). Although there was no significant difference in color, texture, and WHC values between the 2 groups after processing into roasted chicken breast (*P* > 0.05), an approximately 3 % higher cooking loss was observed in PSE group compared to its counterpart (*P* < 0.05). Moreover, cooking loss and lightness values showed a significant positive correlation. Nevertheless, there were no significant differences in sensory parameters between the 2 products (*P* > 0.05).

**Conclusions:**

These results indicated that an economical loss can be expected due to the significantly higher cooking loss observed in roasted breast processed from PSE meat.

## Background

Color is a significant quality parameter that influences the consumer acceptance and selection of both raw and processed meat [1]. Poultry producers go to great lengths to manufacture products with the accurate color and to avoid appearance defects which unfavorably affect product selection or price [2, 3]. Color defects of meat may occur due to several reasons, including the pale, soft, and exudative (PSE) condition. Currently, PSE condition has become a growing problem in the meat industry and results in meat with pale color, low water holding capacity (WHC), and softer texture [4]. A number of pre-slaughter factors [5, 6], stunning methods [4], and chilling regimes [4, 7] are associated with PSE meat formation in broiler chickens.

When used in processed meat products, PSE meat results in products with decreased cooking yield and a dry texture that is undesirable to consumers [4]. Further, it has poor processing characteristics and a greater potential of spoilage compared to normal meat [8]. Woelfel et al. [4] further reported that there is a higher potential for great economic loss in the production of whole muscle products and further processed products such as formed breast loaves and rolls due to the usage of PSE meat.

Chicken meat production in Sri Lanka increased by 4.5 % to 150,980 metric tons in 2014 [9]. However, no scientific literature is available on the prevalence of PSE condition in chicken meat in Sri Lanka. Therefore, this study was conducted to determine the incidence of PSE chicken meat in a commercial meat processing plant and to find out its consequences on the meat quality traits of roasted chicken breast.

## Methods

### Experiment 1

#### Incidence of PSE meat

A total of 10 kilograms (10 ± 0.1 kg) of skinless, boneless, and frozen broiler breast fillets per day were collected from each of 3 different commercial broiler processing plants (A, B, and C) on the day of delivery to the meat processing plant. The experiment was repeated 2 times over 2 different weeks at the same plant as the number of available fillets for each color group may vary according to flock and processing plant conditions [10]. A total of 60 breast fillets (20 breast fillets from each company) were randomly selected, thawed for 30 min after delivery, evaluated based on color, and placed into 1 of 2 categories; paler than normal (PSE) or normal color. Samples were classified as PSE or normal meat samples based on their L* values; PSE: L* > 58 and normal: L* ≤ 58. A total of 20 breast fillets (10 PSE and 10 normal) were then analyzed for pH and WHC.

#### Color values

A Minolta CR-410 colorimeter (Konica Minolta, Japan) calibrated against a white reference tile was used to evaluate the color values at the dorsal surface of the intact skinless breast muscles. The values of lightness (L*), redness (a*), and yellowness (b*) were obtained at 3 sites on the same sample as explained by Carvalho et al. [11]; the proximal extremity of the muscle, the distal extremity, and the between the proximal and the distal extremity and the average value of each sample was used.

#### pH value

For pH measurement, 1 g from each sample which was thawed for 30 min after delivery was homogenized (T25b, Ika Works (Asia), Sdn, Bhd, Malaysia) with 9 mL of distilled water at 1,130 × *g* for 30 sec. Supernatant was then filtered (No. 4; Whatman International Ltd) and pH of filtrate was determined using a pH meter (PH700, Eutech Instrument, Singapore) after calibration using buffers (pH 4.01, 7.00 and 10.01) at room temperature. The mean value of 2 repeated measurements from each sample was used.

#### WHC

WHC was determined based on the technique described by Hamm [12], as described in Wilhelm et al. [13]. A total of 20 samples were analyzed in duplicate. First, samples were cut into cubes of 2.0 ± 0.10 g. They were then carefully placed between 2 pieces of filter papers (No. 4; Whatman International Ltd, Maidstone, England) on acrylic plates and left under a 10-kg weight for 5 min separately. After recording the final weight of each sample, WHC was calculated using the following equation, where *Wi* and *Wf* are the initial and final weights of sample, respectively.

$$ \mathrm{W}\mathrm{H}\mathrm{C}\left(\%\right)=100-\left[\frac{\left(Wi-Wf\right)\times 100}{Wi}\right] $$

### Experiment 2

#### Processing of roasted chicken breast

Breast fillet samples from PSE and normal groups were processed separately into roasted chicken breast at a commercial meat processing plant. First, fillets were mixed with non-meat ingredients; 20 % water, 0.8 % white pepper, 1.6 % soy sauce, 1.4 % vacuum salt, 0.2 % monosodium glutamate and 0.2 % phosphate, and tumbled for 20 min separately. After tumbling, each breast fillet was weighed and oven-roasted at 90 °C for 45 min until a core temperature of 72 °C was obtained. After cooling the fillets to room temperature, they were weighed again. Next, each sample was vacuum packed and frozen at − 30 °C for 1 day. Finally, samples were transported to laboratory under refrigerated condition for further analysis.

#### Cooking loss

The cooking loss was calculated based on the weight loss took place during cooking as a percentage of the initial weight [14].

#### Hardness

Eight samples of the roasted chicken breast (4 from each category) were cut into rectangular pieces (2 cm height × 2 cm width × 2 cm length). Hardness was measured using Guss texture analyzer (South Africa) and the results were expressed in kilograms (kg). The test conditions were as follows: measuring distance 10 mm, measuring speed 10 mm/s, probe diameter 5 mm. Average value of 3 measurements was taken from different locations (from the middle and 2 corners of the sample) of each meat sample.

#### Color, pH and WHC

These assays were similar to those described in the above sections.

### Sensory evaluation

Roasted chicken breasts prepared from PSE and normal meats were cut into 2 cm × 2 cm × 2 cm cubes and heated in a microwave at 105 °C for 5 min separately. All samples were labeled with random 3 digit numbers. Samples were then placed on labeled white plates and served for 30 panelists with drinking water. A 7-point hedonic scale (1 = dislike very much, 7 = like very much) was used to evaluate the sensory parameters. The sensory parameters tested were appearance, color, odor, taste, juiciness, tenderness, and overall acceptability.

### Statistical analysis

Statistical analysis of the results was carried out using a one-way analysis of variance (ANOVA) by the general linear model procedure of SAS program version 9.3 [15]. Mean separation was conducted using Duncan’s multiple range tests at *P* < 0.05. Correlation coefficients between variables were analyzed using the Microsoft Excel 2013.

## Results

This study is the first survey carried out to evaluate the magnitude of color variation in broiler meat used for further processing in Sri Lanka.

### Experiment 1

#### Incidence of PSE meat

**Fig. 1:**
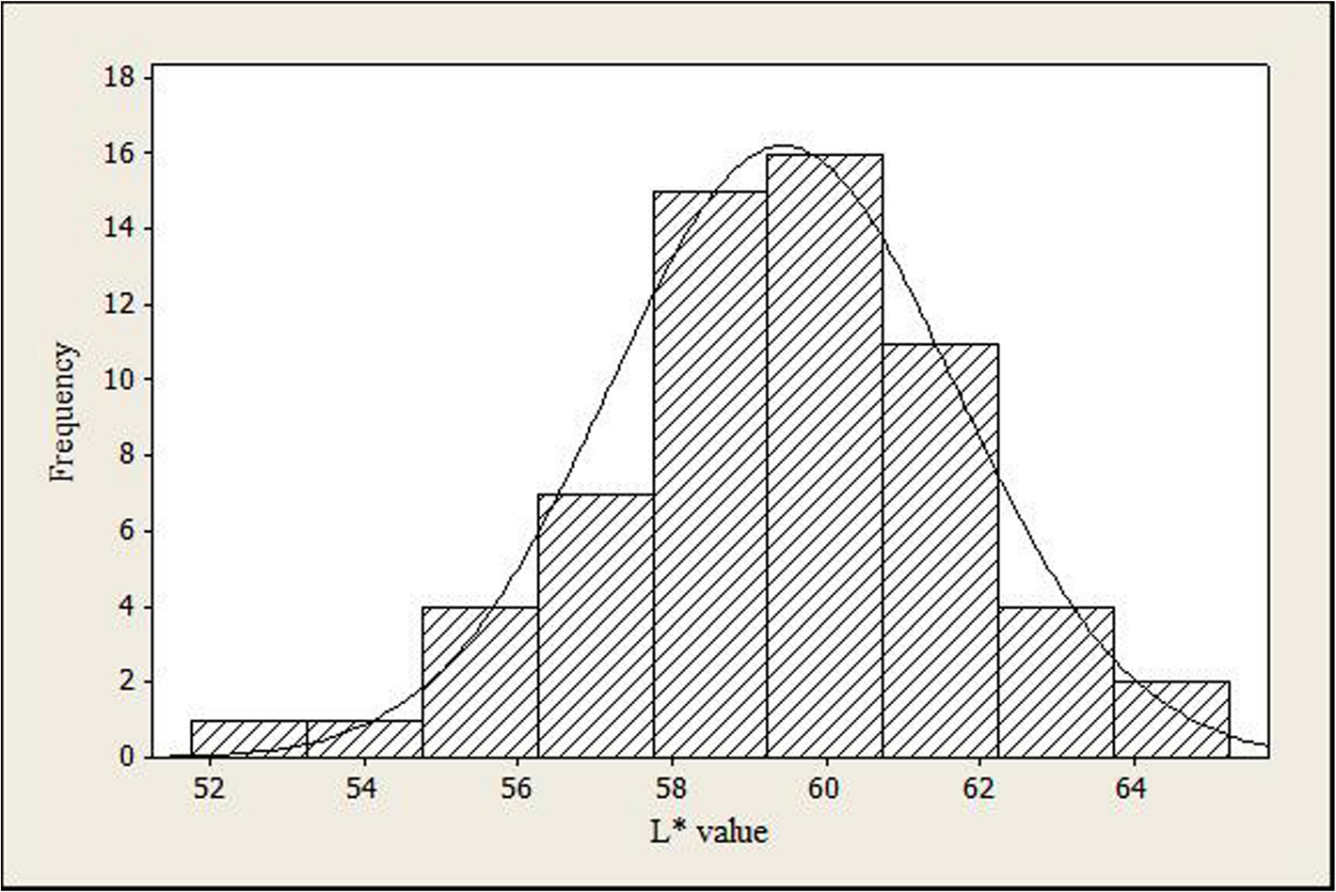
A histogram showing the distribution of L* values in broiler breast fillets (*n =* 60)

A representative histogram obtained for the color measurement of 60 breast meat samples is presented in Fig. 1. The distribution shows a bell shape curve with an average L* value of 59.45. Table 1 shows the incidence of PSE meat among the 3 different poultry processing companies. The highest incidence of PSE meat was reported by company A and the highest color variation was occurred in meat from company C. On average, 70 % of the breast fillets showed PSE condition in this study. Table 2 shows the comparative values of PSE and normal breast fillets observed in different studies in relation to the results described in this work. According to Table 2, the incidence of PSE condition observed in the present study was much higher compared to those reported in Canada, USA, Portugal, Italy, and Brazil.

**Table 1:** Incidence of PSE meat among three different poultry processing companies

	Company A	Company B	Company C	Total
n	20	20	20	60
L* range	56–63	55–61	52–64	52–64
a* range	10–13	9–15	7–14	7–15
b* range	8–16	8–16	9–16	8–16
Incidence %	80	60	70	70

**Table 2:** Comparative color L* values and prevalence of PSE condition in breast fillets observed in different studies

	Barbut [17]	Owens et al. [18]	Fraqueza et al. [35]	Carvalho et al. [11]	Woelfel et al. [4]	Petracci et al. [3]	Present study
Country	Canada	USA	Portugal	Brazil	USA	Italy	Sri Lanka
Species	Turkey	Turkey	Turkey	Turkey	Chicken	Chicken	Chicken
L* range	38–57	41–63	35–55	42–66	42–71	40–66	52–64
L* cutoff	50/51	53	50	53	54	56	58
Incidence %	12	40	8	41.7	47	10	70

#### Color values of raw meat

The results of the color values between PSE and normal meat are presented in Table 3. As expected, a significant difference in lightness values between PSE and normal meat samples was detected (*P* < 0.05). In PSE samples, the average L* value was 4 units higher than that in normal samples. In contrast, a* and b* values of both PSE and normal breast fillets were not significantly different (*P* > 0.05).

**Table 3:** Mean quality attributes of normal and PSE broiler breast fillets

Measurement	Fresh meat samples		SEM^1^
	Normal	PSE	
L* value	56.82^b^	61.83^a^	0.37
a* value	12.43	11.61	0.39
b* value	11.92	11.94	0.36
pH	5.97^a^	5.83^b^	0.02
Water holding capacity (%)	77.95	77.12	0.86

^a,b^Means within each row with different superscripts are significantly different (*P* < 0.05)

^1^Standard error of the means (*n =* 20)

#### pH and WHC values of raw meat

**Fig. 2:**
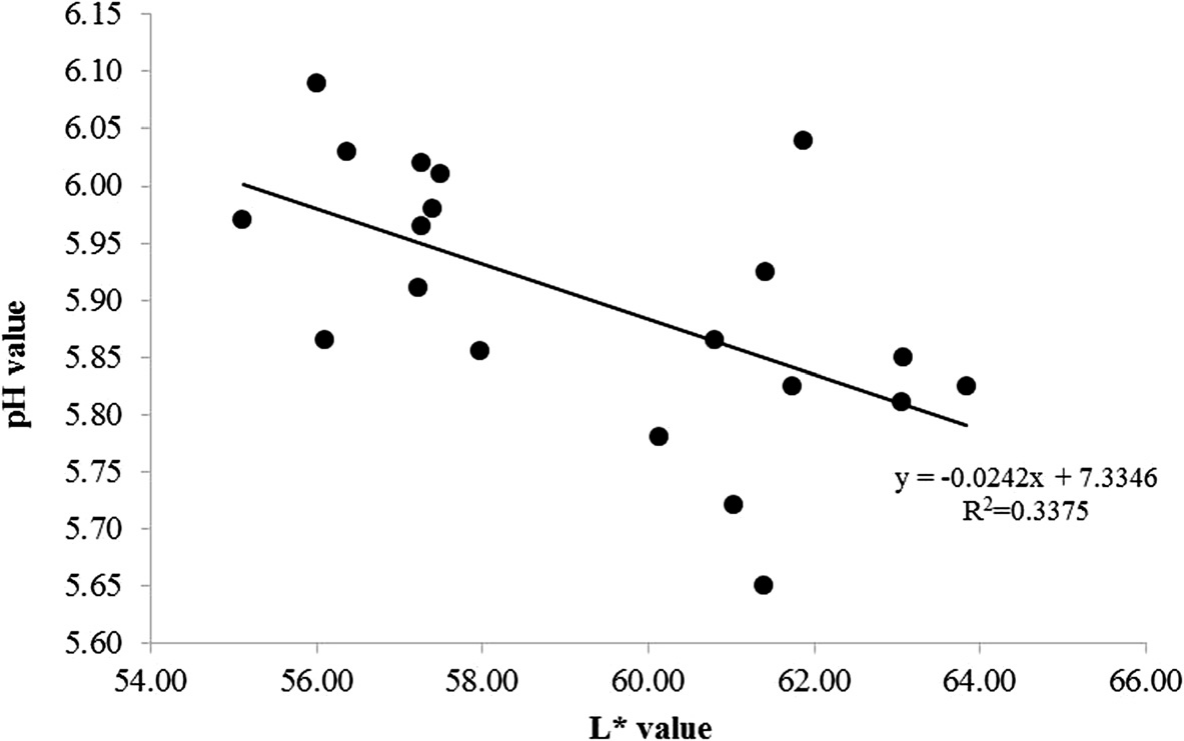
The relationship between L* and pH values in broiler breast fillets (*n =* 20)

Table 3 further shows the pH and WHC values for PSE and normal breast fillets. Normal and PSE breast fillets were significantly different (*P* < 0.05) in pH values; the pH value of PSE samples was significantly lower than that of normal samples. However, differences in WHC were not observed between the PSE and normal meat samples in the current study (*P* > 0.05). Additionally, there was a significant (*P* < 0.05) negative Pearson correlation between the pH and L* values reported in the present study (Fig. 2), with a moderate value of coefficient (-0.58). The L* value increased as muscle pH decreased.

### Experiment 2

#### Color and pH values of roasted chicken breast

Experiment 2 was conducted to find out the effect of PSE broiler meat on functional qualities of 1 of the processed products**—**roasted chicken breast—manufactured using general commercial ingredients and processing conditions. The color and pH values of roasted chicken breast are presented in Table 4 and it showed no significant differences (*P* > 0.05) between the 2 groups regarding the L*, a*, and b* values. In addition, significantly higher pH values were observed in roasted breast from PSE meat than those from normal meat.

**Table 4:** Mean quality attributes of roasted chicken breast processed with PSE and normal meat

Measurement	Roasted chicken breast		SEM^1^
	Normal	PSE	
L*	57.88	57.09	0.69
a*	11.64	12.49	0.32
b*	24.39	24.55	0.38
pH	6.21^b^	6.37^a^	0.01
Water holding capacity (%)	80.85	78.56	1.27
Cooking loss	19.10^b^	22.29^a^	0.63

^a,b^Means within each row with different superscripts are significantly different (*P* < 0.05)

^1^Standard error of the means (*n =* 20)

#### WHC, cooking loss and hardness of roasted chicken breast

**Fig. 3:**
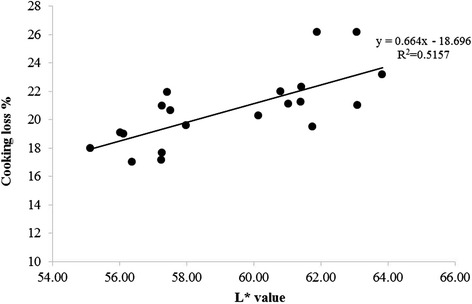
The relationship between L* and cooking loss (%) values in broiler breast fillets (*n =* 20)

**Fig. 4:**
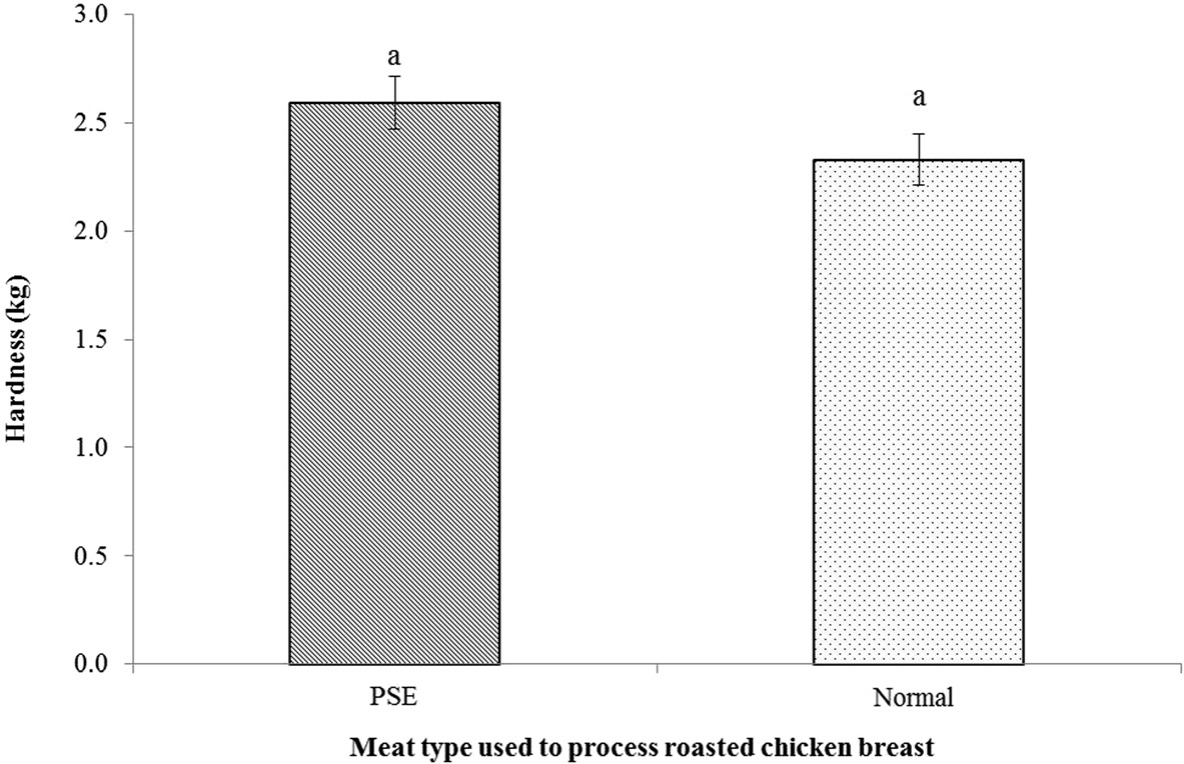
Hardness values (kg) of roasted chicken breast processed with PSE and normal meat (*n =* 20)

Table 4 further presents the results of WHC and cooking loss values of roasted chicken breast made from normal and PSE meats. The WHC values of roasted chicken breast meat had similar trends as those of raw meat; WHC did not differ (*P* > 0.05) between the 2 groups compared in this study. In contrast, cooking loss showed a significant difference between the 2 groups (*P* < 0.05). PSE samples had an average 3 % higher cooking loss compared with normal samples (*P* < 0.05). The L* value was positively correlated with cooking loss as shown in Fig. 3. The lighter meat showed higher cooking losses in the present study. However, there was no significant difference in hardness between the 2 groups tested in the present study as shown in Fig. 4 (*P* > 0.05).

### Sensory characteristics

The results of the sensory analysis of roasted breast processed from PSE and normal meat are shown in Table 5. There were no significant differences in appearance, color, odor, taste, juiciness, tenderness, and overall acceptability between roasted breasts processed from PSE and normal fillets (*P* > 0.05).

**Table 5:** Sensory analysis of roasted chicken breast fillets processed with PSE and normal meat

Parameter	Roasted chicken breast		SEM^a^
	Normal	PSE	
Appearance	5.50	5.26	0.16
Color	5.20	4.96	0.17
Odor	5.13	5.26	0.20
Taste	5.13	5.26	0.20
Juiciness	5.66	5.23	0.17
Tenderness	5.63	5.20	0.17
Overall acceptability	5.60	5.36	0.12

^a^Standard error of the means (*n =* 60)

## Discussion

### Incidence of PSE meat

According to Boulianne and King [16], L* value could be used with high sensitivity and high specificity to differentiate pale samples from normal samples. In addition, number of researches has suggested that lightness values can be used as an indicator of poultry breast meat quality for further processing and for evaluating the prevalence of PSE condition in poultry [3, 4]. Different authors have suggested different cutoff L* values to determine the PSE condition such as 50/51 [17], 53 [18], 56 [3], 57 [19, 20]. Some researchers proposed that the cutoff value should be determined by each research laboratory or commercial plant for more reliable conformation of PSE meat [11, 21]. In the present study, a closer cutoff value to that reported by Wilkins et al. [19] for broiler meat was selected. The difference observed in prevalence of PSE condition and in color variation in the present study (Table 1) can be attributed to different processing plant conditions and flock type [10].

In the present study, the L* value range was different from those reported in Canada, USA, Portugal, Italy, and Brazil for turkey and broilers [3, 4, 11, 17, 18]. The high incidence of PSE meat is most likely the result of combined effects of a high environment temperature and RH, as reported previously for broilers [6, 11, 22, 23].

### Color values of raw meat

The results of the color values between PSE and normal meat used in the present study was in well agreement with those of Petracci et al. [3], Woelfel et al. [4], Barbut [24], and Van Laack et al. [25], who found significantly higher L* values in PSE meat than in normal meat. Similar to our findings, the b* values were not significantly different between normal and pale meat in the studies of Petracci et al. [3] and Fletcher et al. [26]. Antemortem temperature stress and excitement immediately before slaughter have been shown to affect poultry meat color [3].

### pH and WHC values of raw meat

Our finding on significantly lower pH value in PSE breast fillets was consistent with earlier observations regarding turkey and broiler meat [3, 4, 24, 27]. WHC is an important meat quality attribute which can be used to evaluate PSE meat [4]. PSE meat has a lower WHC value due to its low pH and obviously by the consequence of the denaturation of myofibrilar and sarcoplasmic proteins [28]. In addition, Barbut [24] reported that lower muscle pH was associated with lower WHC, as evident in pale turkey meat. In contrast to our findings on WHC, Woelfel et al. [4] detected significant higher expressible moisture and drip loss values in pale broiler meat compared to normal meat.

The relationship between the pH and L* values observed in the current study corresponds with the findings of Carvalho et al. [11] and Barbut [24]. Similarly, Van Hoof [27] reported that the apparent pale color in poultry meat is associated with lower pH and that meat is susceptible to a PSE like condition. In addition, Fletcher [29] reported that variations in color have a strong correlation with muscle pH; darker muscles having higher pH values and lighter muscles having lower pH values. If rigor development is accelerated resulting in lower muscle pH, it is likely that sarcoplasmic and myofibrillar proteins begin to denature resulting in pale meat [18].

### Color and pH values of roasted chicken breast

The results of Fletcher et al. [26] on color values of cooked meat products is not in agreement with those of the present study. They reported that the L* values were significantly different in the cooked meat products. Moreover, Carvalho et al. [11] observed that processed PSE meat had a higher L* value compared to normal fillets. When the L* values of PSE meat in both raw and processed forms are considered, processing had reduced the variation in L* values and the difference between the color groups. Therefore, processing had a favorable effect in reducing the potential negative characteristics of raw PSE meat.

In contrast to our findings, Carvalho et al. [11] reported significantly higher pH values in normal meat than in PSE meat. Daigle et al. [30] reported that the addition of ingredients improves some of the functional properties of turkey chunks. Hence, the addition of phosphate might have increased the pH value of roasted breast processed using PSE meat.

### WHC, cooking loss and hardness of roasted chicken breast

Our findings on WHC of roasted chicken breast are comparable with the findings of Kissel et al. [31] on processed mortadella prepared using PSE and normal broiler meat. Moreover, Daigle et al. [30] observed similar results in delicatessen rolls produced from normal and PSE turkey meat.

Petracci et al. [3] and Zhang and Barbut [32] reported significantly higher cooking loss for PSE breast meat compared to normal breast meat and it is well agreed with the results of the present study. Similarly, Barbut [24] found a 9 % difference in cooking loss between dark and pale turkey samples. The positive correlation observed in the present study between the L* value and cooking loss confirmed the previous observation of Barbut [24] who suggested that L* value was highly correlated with cooking loss. Cooking loss represents 1 of the most important parameters in the industry, which seeks for raw materials with high moisture retention ability and it is especially important in whole breast muscle products such as oven roasted/ smoked breast [24].

Hardness is 1 of the most important attributes in consumers’ final satisfaction on poultry meat [2]. Barbut [24] reported that meat with poor WHC resulted in dryer and tougher meat. In the current study, we did not observe any difference in WHC and this might be the reason that no significant difference in hardness between the 2 samples was detected. Similarly, Petracci et al. [3] observed comparable shear values between normal and pale broiler meat.

### Sensory characteristics

The results of the sensory analysis of roasted breast processed from PSE and normal meat were comparable to the findings of Garcia et al. [33] who observed no significant differences in the sensorial attributes of PSE breast fillets compared with normal fillets. Sensorial characteristics such as color, taste, juiciness, and tenderness partially depend on WHC [34]. The present study showed no significant difference in WHC between normal and PSE meats before and after processing. In addition, no significant difference in texture of roasted chicken breasts processed from PSE and normal meat was reported. Therefore, these might be the reasons that no significant differences in sensory characteristics were detected between PSE and normal groups.

## Conclusions

The incidence of PSE meat found in the present study was 70 % and it was higher than those reported in previous studies. These results suggest that a large portion of commercially processed broiler meat can be pale in color and has the potential for lower pH value. However, there were no difference in color, WHC, and texture when fillets were processed into roasted chicken breast (*P* > 0.05). An approximately 3 % higher cooking loss was reported for roasted chicken breast processed from PSE meat compared to those processed from normal meat, and this may lead to an economical loss in processing industry. These results may not represent the entire industry but indicate that PSE chicken can represent a substantial proportion of broiler chicken meat used for further processing.

## Abbreviations

PSE, pale, soft, exudative; WHC, water holding capacity
